# Soluble Immune Checkpoint Molecules as Predictors of Efficacy in Immuno-Oncology Combination Therapy in Advanced Renal Cell Carcinoma

**DOI:** 10.3390/curroncol31040129

**Published:** 2024-03-22

**Authors:** Kosuke Ueda, Keiichiro Uemura, Naoki Ito, Yuya Sakai, Satoshi Ohnishi, Hiroki Suekane, Hirofumi Kurose, Tasuku Hiroshige, Katsuaki Chikui, Kiyoaki Nishihara, Makoto Nakiri, Shigetaka Suekane, Sachiko Ogasawara, Hirohisa Yano, Tsukasa Igawa

**Affiliations:** 1 Department of Urology, Kurume University School of Medicine, Kurume 830-0011, Japan; uemura_keiichirou@kurume-u.ac.jp (K.U.); itou_naoki@kurume-u.ac.jp (N.I.); sakai_yuuya@kurume-u.ac.jp (Y.S.); ohnishi_satoshi@med.kurume-u.ac.jp (S.O.); suekane_hiroki@med.kurume-u.ac.jp (H.S.); kurose_hirofumi@med.kurume-u.ac.jp (H.K.); hiroshige_tasuku@med.kurume-u.ac.jp (T.H.); chikui_katsuaki@med.kurume-u.ac.jp (K.C.); nishihara_kiyoaki@kurume-u.ac.jp (K.N.); mnakiri@med.kurume-u.ac.jp (M.N.); suekane@med.kurume-u.ac.jp (S.S.); tigawa@med.kurume-u.ac.jp (T.I.); 2 Department of Pathology, Kurume University School of Medicine, Kurume 830-0011, Japan; sachiko@med.kurume-u.ac.jp (S.O.); hiroyano@kurume-u.ac.jp (H.Y.)

**Keywords:** soluble immune checkpoint molecules, renal cell carcinoma, immune checkpoint inhibitor

## Abstract

Immuno-oncology (IO) combination therapy is the first-line treatment for advanced renal cell carcinoma (RCC). However, biomarkers for predicting the response to IO combination therapy are lacking. Here, we investigated the association between the expression of soluble immune checkpoint molecules and the therapeutic efficacy of IO combination therapy in advanced RCC. The expression of soluble programmed cell death-1 (sPD-1), soluble programmed cell death ligand-1 (sPD-L1), soluble PD-L2 (sPD-L2), and lymphocyte activation gene-3 (sLAG-3) was assessed in plasma samples from 42 patients with advanced RCC who received first-line IO combination therapy. All IMDC risk classifications were represented among the patients, including 14.3, 57.1, and 28.6% with favorable, intermediate, and poor risk, respectively. Univariate analysis revealed that prior nephrectomy, sPD-L2 levels, and sLAG-3 levels were significant factors affecting progression-free survival (PFS), whereas multivariate analyses suggested that sPD-L2 and sLAG-3 levels were independent prognostic factors for PFS. In a univariate analysis of the overall survival, prior nephrectomy and sPD-L2 levels were significant factors; no significant differences were observed in the multivariate analysis. No significant correlation was observed between the sPD-L2 and sLAG-3 levels and PD-L2 and LAG-3 expression via immunohistochemistry. In conclusion, sPD-L2 and sLAG-3 expression may serve as a potential biomarker for predicting IO combination therapy efficacy.

## 1. Introduction

Immune checkpoint inhibitors (ICIs) targeting programmed cell death-1 (PD-1), programmed cell death ligand-1 (PD-L1), and cytotoxic T-lymphocyte-associated antigen 4 (CTLA-4) are a major class of immuno-oncology therapeutics that have significantly improved the prognosis of advanced renal cell carcinoma (RCC) [1]. Following clinical trials, immuno-oncology (IO) combination therapies such as IO + IO therapy or IO therapy + tyrosine kinase inhibitors (TKIs) have become the standard first-line treatment for advanced RCC in Japan [2,3,4,5].

In recent years, a variety of biomarkers have been investigated to reflect the effects of ICIs, including PD-L1 expression in tumor cells, the tumor mutation burden, the neoantigen burden, polybromo1 gene mutation, immune cell infiltration, and the gut microbiota. However, biomarkers for the efficacy of IO combination therapy remain elusive. Therefore, the search for biomarkers that predict the effect of IO combination therapy is required for patients with advanced RCC.

Several studies have reported that PD-L1 expression in RCC tumor cells is associated with prognosis and the therapeutic response to TKIs [6,7]. In contrast, high PD-L1 expression has been shown to be associated with better therapeutic effects than low PD-L1 expression in patients with RCC treated with immune checkpoint inhibitors [1]. Furthermore, several studies have indicated that PD-L1 expression, programmed cell death-ligand 2 (PD-L2), and lymphocyte activation gene-3 (LAG-3) are associated with the therapeutic efficacy of ICI treatment for advanced RCC [7,8,9,10]. However, these reports are controversial, indicating the limited utility of PD-L1 as a predictive biomarker in RCC and that treatment decisions in RCC should not depend on PD-L1 expression levels [11].

In recent years, soluble immune checkpoint molecules released into the blood after the cleavage of the extracellular domain of tumor cells have attracted attention [12,13]. Transmembrane PD-L1 has a soluble form that is produced by tumor cells or activated mature dendritic cells and is associated with diversity in the composition and function of the PD-1/PD-L1 signaling pathway [14]. Soluble PD-L1 (sPD-L1) appears to decrease interferon-gamma secretion by T cells and may be involved in systemic antitumor immunomodulation by targeting T lymphocytes in secondary lymphoid organs [15]. The relationship between the expression of sPD-L1 and the prognosis of advanced RCC is unclear. In addition, there are few reports on the clinical role of soluble immune checkpoint molecules such as sPD-L2 and sLAG-3 in advanced RCC.

Here, we investigated the potential roles of new plasma biomarkers, including soluble PD-1 (sPD-1), sPD-L1, soluble PD-L2 (sPD-L2), and soluble LAG-3 (sLAG-3), as putative predictive biomarkers for determining the efficacy of IO combination therapy in advanced RCC. In addition, we also examined the association between the soluble immune checkpoint molecule levels and the expression of immune checkpoint molecules in tumors.

## 2. Materials and Methods

### 2.1. Patients

This study included 42 patients who had received IO combination therapies such as nivolumab plus ipilimumab (NIVO + IPI), axitinib plus avelumab (AXI + AVEL), and lenvatinib plus pembrolizumab (LEN + PEMB) as first-line treatment at the Kurume University Hospital from July 2020 to August 2022. Pretreatment assessments of the patients’ clinical characteristics and blood data were performed immediately before the initiation of IO combination therapy. This study was conducted in full compliance with the Declaration of Helsinki of the World Medical Association and was approved by the Ethics Review Committee of the Kurume University School of Medicine (approval number: 20118). Written informed consent was obtained from all patients before their participation in this study. The clinical information of these patients was obtained from their medical records and retrospectively reviewed and analyzed.

### 2.2. ELISA

Following enrollment, peripheral blood samples were collected from the patients in tubes containing heparin as an anticoagulant before the initiation of first-line treatment. The sPD-1, sPD-L1, sPD-L2, and sLAG-3 concentrations were calculated using an enzyme-linked immunosorbent assay (ELISA). ELISA tests were performed using commercial kits (ab252360 H9uman PD-1 ELISA Kit, Abcam; ab214565 Human PD-L1 (clone 28-8) ELISA Kit, Abcam, Cambridge, UK; ab231928 Human PD-L2 ELISA Kit, Abcam, Cambridge, UK; and ab193707 Human LAG-3 ELISA Kit, Abcam, Cambridge, UK) according to the manufacturer’s instructions. To generate standard curves, the recombinant protein corresponding to each test was used at a prespecified concentration. The results were obtained using a spectrophotometer (absorbance at 450 nm). Additionally, the concentrations were calculated based on standard curves. All samples, standards, and negative controls were analyzed in duplicate.

### 2.3. Immunohistochemical Analysis

Paraffin-embedded tissue samples were cut to a thickness of 4 µm, placed on a glass slide for examination, and labeled with either anti-PD-L2 antibodies (1:1000; clone 176611, R&D Systems, Minneapolis, MN, USA) or anti-LAG-3 antibodies (1:100; clone 17B4, Novus Biologicals, CO, USA) using a BenchMark ULTRA (Ventana Automated Systems, Inc., Tucson, AZ, USA). As previously described, PD-L2 expression in tumors was considered positive if ≧5% of tumor cells were present [7]. Similarly, LAG-3 expression in tumors was positive if ≧1% of tumor cells were present [16].

### 2.4. Statistical Analysis

Progression-free survival (PFS) for first-line IO combination therapy and overall survival (OS) from the initiation of IO combination therapy to the date of mortality was determined using the Kaplan–Meier method, and analyzed using the log-rank test. A comparison of PFS and OS between and among cohorts was achieved via a log-rank test. Univariate and multivariate analyses using the Cox proportional hazards model were performed to identify the risk factors for PFS and OS based on the calculation of hazard ratios (HR) with 95% confidence intervals (CI). The median values of sPD-1, sPD-L1, sPD-L2, and sLAG-3 were used as the cutoff values. All statistical analyses were performed using JMP version 17 (SAS Institute, Inc., Cary, NC, USA) and a value of *p* < 0.05 was considered.

## 3. Results

### 3.1. Patient Characteristics

The patient characteristics are summarized in Table 1. The median age of the participants was 69.5 years (range, 42–80 years), and the majority of patients were male (81.0%). All international metastatic renal cell carcinoma database consortium (IMDC) risk groups were represented among the patients, with 14.3, 57.1, and 28.6% of the patients presenting a favorable, intermediate, and poor risk, respectively. The percentage of patients who underwent nephrectomy before IO combination therapy was 45.2%. The majority of the patients were diagnosed with advanced RCC with clear cell histology (81.0%). Most patients received NIVO + IPI (61.9%) or AXI + AVEL (31.0%) as first-line IO combination therapy. The remaining patients were treated with LEN + PEMB.

The levels of soluble immune checkpoint molecules before the initiation of IO combination therapy are shown in Table 1. The median sPD-1, sPD-L1, sPD-L2, and sLAG-3 concentrations were 291.3 pg/mL (149.4–1649.9), 39.8 pg/mL (8.0–395.8), 9291.6 (1726.1–27,900.8) pg/mL, and 19.4 ng/mL (15.2–41.1), respectively. The median pretreatment C-reactive protein and neutrophil to lymphocyte ratio were 0.71 mg/dL and 3.28, respectively.

### 3.2. Baseline Soluble Immune Checkpoint Molecules as Predictive Biomarkers of IO Combination Therapy Outcome in Advanced RCC

**Figure 1:**
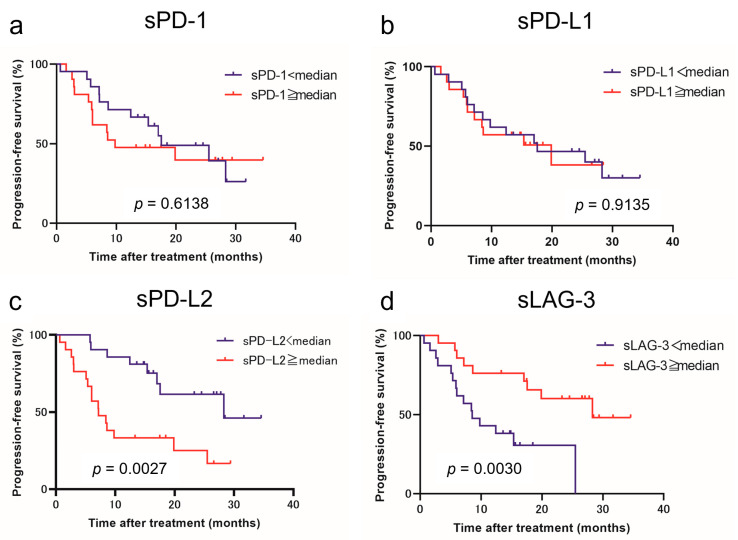
Progression-free survival (PFS) in patients with advanced renal cell carcinoma treated with immuno-oncology combination therapy according to the pretreatment soluble immune checkpoint molecules. Survival curve PFS with respect to sPD-1 (**a**), sPD-L1 (**b**), sPD-L2 (**c**), and sLAG-3 (**d**) levels.

**Figure 2:**
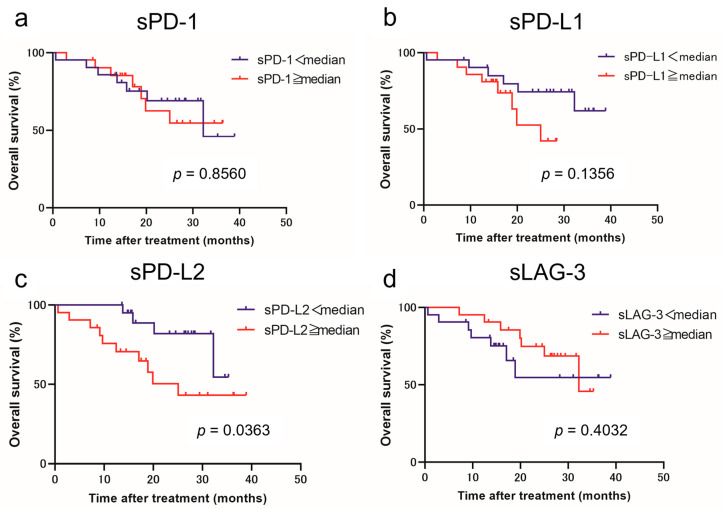
Overall survival (OS) in patients with advanced renal cell carcinoma treated with immuno-oncology combination therapy according to the pretreatment soluble immune checkpoint molecule levels. Survival curve OS with respect to sPD-1 (**a**), sPD-L1 (**b**), sPD-L2 (**c**), and sLAG-3 (**d**) levels.

Using the cutoff values determined from the median values, we classified patients with low and high levels of each immune checkpoint molecule. Figure 1 and Figure 2 show the estimated PFS and OS curves of patients with advanced RCC treated with IO combination therapy according to their sPD-1, sPD-L1, sPD-L2, and sLAG-3 levels, respectively. A high sPD-L2 level was a predictor of significantly worse PFS (*p* = 0.0027) and OS (*p* = 0.0363) than a low sPD-L2 level. On the other hand, a low sLAG-3 level was a predictor of significantly worse PFS (*p* = 0.0030) than a high sLAG-3 level. However, no significant difference in OS was observed between the two groups. In contrast, no significant differences in PFS and OS were observed between patients with low and high sPD-1 and sPD-L1 levels.

### 3.3. Soluble Immune Checkpoint Molecules Expression and Clinical Course

To identify the pretreatment prognostic factors before IO combination therapy associated with PFS and OS, we performed univariate and multivariate analyses using the Cox proportional hazards model (Table 2 and Table 3). The univariate analysis revealed that prior nephrectomy (HR = 2.659, 95% CI = 1.102–6.412, *p* = 0.0295), sPD-L2 levels (HR = 3.455, 95% CI = 1.463–8.158, *p* = 0.0047), and sLAG-3 levels (HR = 0.270, 95% CI = 0.108–0.675, *p* = 0.0051) were significant factors affecting PFS. Multivariate analyses suggested that sPD-L2 (HR = 2.918, 95% CI = 1.201–7.085, *p* = 0.0180) and sLAG-3 (HR = 0.387, 95% CI = 0.154–0.974, *p* = 0.0438) levels had independent prognostic effects on PFS. In the univariate analysis related to OS, prior nephrectomy (HR = 3.768, 95% CI = 1.049–13.533, *p* = 0.0420) and sPD-L2 levels (HR = 3.241, 95% CI = 1.012–10.375, *p* = 0.0477) were the significant factors affecting OS. However, the multivariate analysis showed a trend for prior nephrectomy and sPD-L2, but did not identify an independent factor for OS.

### 3.4. Association between Soluble Immune Checkpoint Molecule Levels and the Expression of Immune Checkpoint Molecules in Tumors

**Figure 3:**
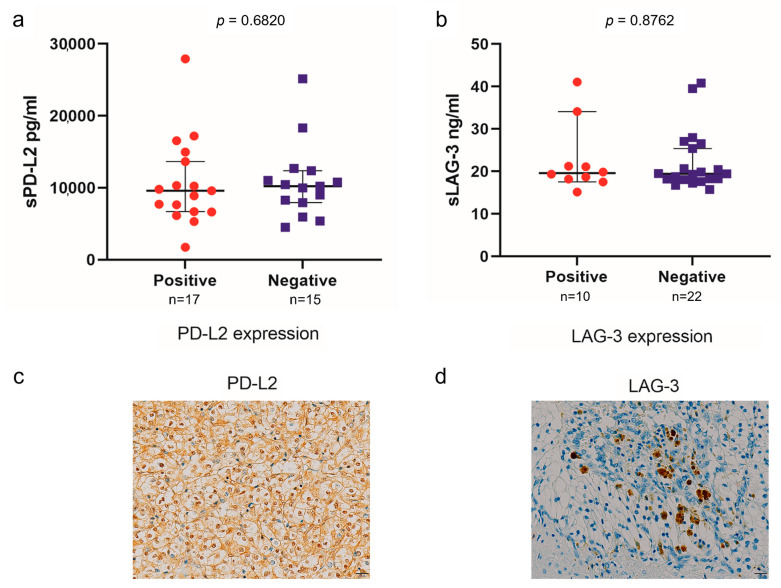
Association between sPD-L2 and PD-L2 expression in tumors (**a**). Association between sLAG-3 and LAG-3 expression in tumors (**b**). The representative immunohistochemical picture of PD-L2 expression in tumors (**c**). The representative immunohistochemical picture of LAG-3 expression in tumors (**d**).

**Figure 4:**
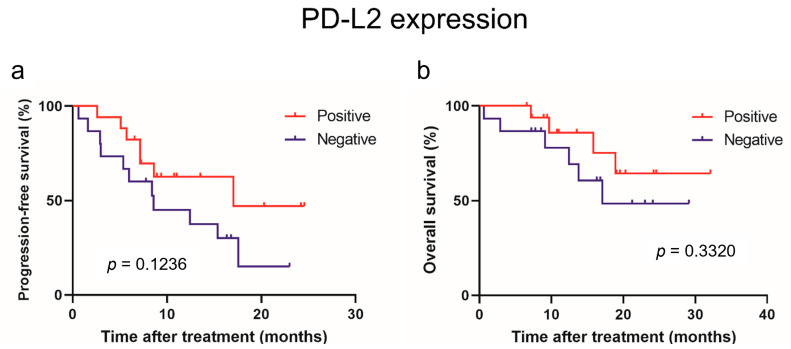
Progression-free survival (**a**) and overall survival (**b**) in patients with advanced renal cell carcinoma treated with mmune-oncology combination therapy based on immunohistochemical expression of PD-L2.

**Figure 5:**
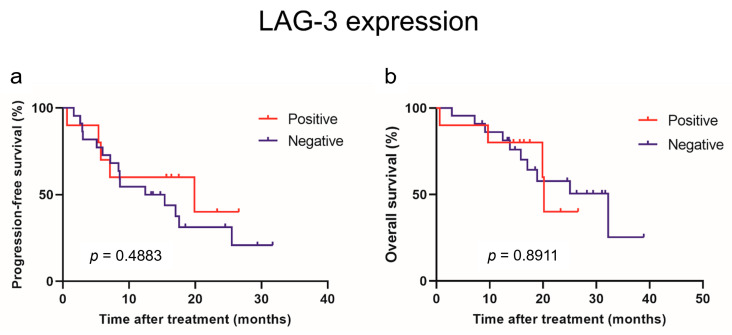
Progression-free survival (**a**) and overall survival (**b**) in patients with advanced renal cell carcinoma treated with mmune-oncology combination therapy based on immunohistochemical expression of LAG-3.

The PD-L2 and LAG-3 expression in tumors was evaluated to investigate their correlation with sPD-L2 and sLAG-3 levels before the initiation of IO combination therapy (Figure 3). No correlation was observed between the sPD-L2 levels and PD-L2 expression in the tumors (*p* = 0.6820). Similarly, no correlation was found between the sLAG-3 levels and LAG-3 expression in tumors (*p* = 0.8762). A survival comparison of the PD-L2 and LAG-3 expression in tumors showed no correlation between expression and PFS or OS (Figure 4 and Figure 5).

## 4. Discussion

PD-1 and its ligands have been reported to exist in soluble forms that can be released by immune and tumor cells and measured in body fluids, such as plasma [17]. There have been several reports on the soluble immune checkpoint molecules in patients with cancer treated with ICIs. However, the clinical roles of these proteins remain controversial.

In recent years, sPD-L1 has also been used as a prognostic biomarker for various cancers [15,18]. It has been reported that sPD-L1 acts as a decoy and attenuates the ICI effects. Furthermore, previous reports have shown that sPD-L1 in the plasma of patients with non-small cell lung cancer (NSCLC) binds to PD-1 and that sPD-L1 may compete with ICIs by binding the PD-1 expressed on the surface of T cells, thereby promoting the exhaustion of activated T cells. Therefore, high sPD-L1 levels may be associated with ICI ineffectiveness. Several studies have shown that high sPD-L1 levels are closely associated with a poor prognosis [13].

Montemagno et al. reported that a high sPD-L1 level is an independent prognostic factor in patients with metastatic RCC treated with sunitinib [19]. Wakita et al. also showed that elevated levels of sPD-L1 may indicate a worse treatment response to nivolumab in metastatic RCC treated with ICIs [20]. However, they showed that patients with low levels of sPD-L1 had a poorer OS than those with high levels of sPD-L1, whereas there was no significant difference in the PFS upon nivolumab treatment. In this study, there was no significant difference in the PFS and OS after IO combination therapy based on sPD-L1 expression. Previous reports have also shown no prognostic value for advanced RCC treated with ICIs based on sPD-L1 levels, similar to our findings. Therefore, further studies are required to better understand the role of sPD-L1 in advanced RCC.

In one study, immunohistochemical analysis revealed significantly lower PD-L2 expression in RCC patients than in patients without PD-L2 expression [7]. In contrast, Shin et al. showed that the expression of PD-L2 in tumors was not associated with VEGF-TKI responsiveness or patient outcomes [21]. Thus, the role of PD-L2 in RCC remains unclear. sPD-L2 is a splice variant of membrane-bound PD-L2 that retains the ability to bind to membrane-bound PD-1 receptors for immune regulation. Few studies have examined the clinical role of sPD-L2 in cancer. Wang et al. showed that elevated preoperative serum sPD-L2 levels are associated with the risk of recurrence in clear cell RCC [22]. Research on patients with NSCLC treated with nivolumab has also shown that low sPD-L2 levels are associated with grade 3–4 toxicities [23]. Recently, several studies have shown that the presence of immune-related adverse events (irAEs) during ICI treatment is a predictor of therapeutic effects [24]. Although there have been no previous reports evaluating sPD-L2 levels and the treatment response to ICIs in patients with advanced RCC, the results of this study suggest that low sPD-L2 levels might be a biomarker able to predict a favorable response in patients treated with ICIs. In our study, univariate analysis showed that prior nephrectomy, sPD-L2 levels, and sLAG-3 levels were significant factors affecting PFS. Multivariate analyses suggested that the sPD-L2 level had an independent prognostic effect on PFS. In the univariate analysis of OS, prior nephrectomy and sPD-L2 levels were significant factors. However, the multivariate analysis did not identify sPD-L2 as an independent predictor of OS.

LAG-3 is a transmembrane protein that is structurally similar to CD4 and is expressed on the surface of activated T cells [25]. Although the role of LAG-3 has not been clarified, it is believed to be involved in the inhibition of T cell proliferation and activation, and is expressed on tumor-infiltrating T cells, T cells exhausted by chronic infection, and regulatory T cells [26]. It is believed to play an important role as an immunosuppressive factor in cancer immunity, infection immunity, autoimmune diseases, and immune checkpoint molecules, such as PD-1 and CTLA-4. In studies on NSCLC, low levels of s-LAG3 were reported to be associated with locally advanced or metastatic disease spread [27]. In breast and gastric cancers, detectable levels of sLAG-3 were associated with a favorable prognosis [28,29]. In contrast, Botticelli et al. demonstrated that sLAG-3 is associated with poor prognosis in head and neck squamous cell carcinoma [30]. Previous reports have not clarified the relationship between sLAG-3 and cancer prognosis. On the other hand, high sLAG-3 levels showed better PFS in this study, while no correlation with OS was observed. Thus, further studies are required to clarify these contradictory findings.

In this study, no correlation was found between sPD-L2 and sLAG-3 concentrations and PD-L2 and LAG-3 expression in the immunohistochemical analysis of renal tumor tissue. Previous reports have shown that the expression of soluble immune checkpoint molecules in the plasma reflects heterogeneity across tumors, whereas the expression of PD-L1 in tumors is heterogeneous, not only within a single tumor site but also between various tumor sites [31,32]. Furthermore, soluble immune checkpoint molecules can be secreted by cells other than tumor cells, such as immune cells [33]. The difference between soluble immune checkpoint molecules’ concentrations and immunohistochemical expression could be explained by the fact that plasma reflects the entire tumor heterogeneity, whereas the expression of immune checkpoint molecules in immunohistochemistry can be heterogeneous within one tumor site as well as between different tumor sites. In our study, there was no significant correlation between the PD-L2 or LAG-3 expression in tumors and the PFS or OS in patients with advanced RCC treated with IO combination therapy.

Our study had several limitations, including its single-institute retrospective design and small sample size. In addition, a standardized cutoff value for the expression of soluble immune checkpoint molecules has not yet been clearly established. Furthermore, our study included patients treated with IO + IO and IO + TKIs as first-line treatments. IO combination therapies (IO + IO and IO + TKI) comprise a combination of different types of agents with different mechanisms of action, and it is debatable whether it is correct to compare these regimens in parallel. Finally, the expressions of PD-L2 and LAG-3 were assessed by radical nephrectomy or renal biopsy. Several reports have shown that, given the significant tumor heterogeneity in RCC, nephrectomy specimens may not accurately reflect the biology of the metastatic sites.

## 5. Conclusions

In conclusion, sPD-L2 and sLAG-3 expression may prove to be a potential biomarker for predicting the therapeutic efficacy of IO combination therapy in advanced RCC. Additionally, sPD-L2 expression may be a prognostic factor for advanced RCC in the era of IO combination therapy. The evaluation of immune checkpoint molecules is minimally invasive and repeatable using blood samples, and thus immune checkpoint molecules could be useful biomarkers for patients with advanced RCC starting first-line treatment. In this scenario, we expect that further research will help to clarify the potential role of soluble immune checkpoint molecules in advanced renal cell carcinoma and assist in the ongoing process of discovering and validating new biomarkers to better predict the response of patients to IO combination therapy. Therefore, additional studies focusing on improving patient survival and the efficacy of IO combination therapy are required in order to validate our findings.

## Figures and Tables

**Table 1:** Clinical and pathological features of patients with advanced renal cell carcinoma treated with immuno-oncology combination therapy.

Variable		All (*n* = 42)
Age (years, range)		69.5 (42–80)
Sex, *n* (%)	Male	34 (81.0)
	Female	8 (19.0)
Histopathology, *n* (%)	Clear cell RCC	34 (81.0)
	Non-clear cell RCC	5 (11.9)
	Unknown	3 (7.1)
Performance status, *n* (%)	0, 1	39 (92.9)
	≥2	3 (7.1)
Prior nephrectomy, *n* (%)	Presence	19 (45.2)
	Absence	23 (54.8)
IMDC risk classification, *n* (%)	Favorable	6 (14.3)
	Intermediate	24 (57.1)
	Poor	12 (28.6)
First line treatment, *n* (%)	Nivolumab plus ipilimumab	26 (61.9)
	Axitinib plus avelumab	13 (31.0)
	Lenvatinib plus pembrolizumab	3 (7.1)
sPD-1, pg/mL, median (range)		291.3 (149.4–1649.9)
sPD-L1, pg/mL, median (range)		39.8 (8.0–395.8)
sPD-L2, pg/mL, median (range)		9291.6 (1726.1–27,900.8)
sLAG-3, ng/mL, median (range)		19.4 (15.2–41.1)
CRP, mg/dl, median (range)		0.71 (0.04–18.58)
NLR, median (range)		3.28 (1.44–12.04)

IMDC, international metastatic renal cell carcinoma database consortium; CCRCC, clear cell renal cell carcinoma; CRP, C-reactive protein; NLR, neutrophil to lymphocyte ratio; sPD-1, soluble programmed cell death-1; sPD-L1, programmed cell death ligand-1; sPD-L2, programmed cell death ligand-2; sLAG-3, soluble lymphocyte activation gene-3.

**Table 2:** Univariate and multivariate analyses of progression-free survival in patients with advanced renal cell carcinoma, treated with immuno-oncology combination therapy.

	Progression-Free Survival (*n* = 42)			
	Univariate Analysis		Multivariate Analysis	
Variable	HR	*p*-Value	HR	*p*-Value
Age (≥70 years)	0.577 (0.252–1.322)	0.1936		
Sex (female)	1.241 (0.460–3.347)	0.6699		
Prior nephrectomy (no)	2.659 (1.102–6.412)	0.0295	2.337 (0.922–5.926)	0.0737
Performance status (≥2)	0.514 (0.069–3.837)	0.5166		
IMDC risk classification (intermediate)	1.291 (0.370–4.510)	0.6886		
(poor)	1.602 (0.411–6.250)	0.4975		
Histology (Non-CCRCC)	0.894 (0.264–3.026)	0.8564		
CRP (mg/dL) (≥median)	1.802 (0.789–4.113)	0.1620		
NLR (≥median)	0.838 (0.375–1.874)	0.6676		
sPD-1 (≥median)	1.229 (0.551–2.741)	0.6148		
sPD-L1 (≥median)	1.046 (0.463–2.361)	0.9136		
sPD-L2 (≥median)	3.455 (1.463–8.158)	0.0047	2.918 (1.201–7.085)	0.0180
sLAG-3 (≥median)	0.270 (0.108–0.675)	0.0051	0.387 (0.154–0.974)	0.0438

IMDC, international metastatic renal cell carcinoma database consortium; CCRCC, clear cell renal cell carcinoma; CRP, C-reactive protein; NLR, neutrophil to lymphocyte ratio; sPD-1, soluble programmed cell death-1; sPD-L1, programmed cell death ligand-1; sPD-L2, programmed cell death ligand-2; sLAG-3, soluble lymphocyte activation gene-3; HR, hazard ratio.

**Table 3:** Univariate and multivariate analyses of overall survival in patients with advanced renal cell carcinoma, treated with immuno-oncology combination therapy.

	Overall Survival (*n* = 42)			
	Univariate Analysis		Multivariate Analysis	
Variable	HR	*p*-Value	HR	*p*-Value
Age (≥70 years)	0.375 (0.117–1.196)	0.0974		
Sex (female)	1.093 (0.302–3.958)	0.8920		
Prior nephrectomy (no)	3.768 (1.049–13.533)	0.0420	3.544 (0.982–12.788)	0.0532
Performance status (≥2)	0.855 (0.111–6.614)	0.8807		
IMDC risk classification (intermediate)	1.952 (0.240–15.878)	0.5319		
(poor)	4.450 (0.529–37.446)	0.1695		
Histology (non-CCRCC)	0.727 (0.094–5.616)	0.7601		
CRP (mg/dL) (≥median)	3.036 (0.947–9.729)	0.0617		
NLR (≥median)	0.864 (0.299–2.494)	0.7869		
sPD-1 (≥median)	1.102 (0.386–3.148)	0.8561		
sPD-L1 (≥median)	2.330 (0.745–7.290)	0.1460		
sPD-L2 (≥median)	3.241 (1.012–10.375)	0.0477	3.040 (0.944–9.793)	0.0625
sLAG-3 (≥median)	0.635 (0.218–1.855)	0.4068		

IMDC, international metastatic renal cell carcinoma database consortium; CCRCC, clear cell renal cell carcinoma; CRP, C-reactive protein; NLR, neutrophil to lymphocyte ratio; sPD-1, soluble programmed cell death-1; sPD-L1, programmed cell death ligand-1; sPD-L2, programmed cell death ligand-2; sLAG-3, soluble lymphocyte activation gene-3.

## Data Availability

The datasets used and/or analyzed during the current study are available from the corresponding author upon reasonable request.
